# Insights into Pediatric *GATA2*-Related MDS: Unveiling Challenges in Clinical Practice

**DOI:** 10.3390/biomedicines13040827

**Published:** 2025-03-30

**Authors:** Andra Daniela Marcu, Ana Maria Bica, Cristina Georgiana Jercan, Letitia Elena Radu, Andreea Nicoleta Serbanica, Dumitru Jardan, Andrei Colita, Simona Olimpia Dima, Ciprian Tomuleasa, Alina Daniela Tanase, Anca Colita

**Affiliations:** 1 Faculty of Medicine, University of Medicine and Pharmacy Carol Davila, 020021 Bucharest, Romania; 2 Pediatric Bone Marrow Transplantation Unit, Fundeni Clinical Institute, 022328 Bucharest, Romania; 3 Molecular Biology Laboratory, MedLife, 010719 Bucharest, Romania; 4 Department of Hematology, Coltea Hospital, 030167 Bucharest, Romania; 5 Center of Excellence in Translational Medicine, Fundeni Clinical Institute, 022328 Bucharest, Romania; 6 Faculty of Medicine, Iuliu Hatieganu University of Medicine and Pharmacy, 400347 Cluj-Napoca, Romania; 7 Department of Hematology, Ion Chiricuta Oncology Institute, 400015 Cluj-Napoca, Romania; 8 Department of Bone Marrow Transplantation, Fundeni Clinical Institute, 022328 Bucharest, Romania

**Keywords:** *GATA2* mutation, children, *GATA2*-related MDS, hematopoietic stem cell transplantation

## Abstract

**Background:** *GATA2*-related myelodysplastic syndrome (*GATA2*-MDS) is a unique predisposition syndrome with a high risk of leukemic transformation. This systematic review synthesizes current literature and presents two illustrative pediatric *GATA2*-MDS cases. **Methods:** Data retrieval from eight cohort and case–control studies provides comprehensive analysis on disease features, diagnostic complexities, management, and outcomes related to hematopoietic stem cell transplantation (HSCT) in *GATA2*-related myeloid malignancies. Additionally, two pediatric cases are included to exemplify clinical and therapeutic challenges in real-world setting. **Results:** The literature data demonstrates high incidence of monosomy 7, and recurrent infections as the most common clinical feature, followed by immunodeficiency and lymphedema. Prognosis clearly worsens with age and HSCT remains the only curative treatment. *GATA2* patients undergoing HSCT experience high rates of graft versus host disease (GvHD) as well as unique neurological, thrombotic, and infectious complications. Transplant-related mortality (TRM) is linked to GvHD and infections. Post-transplant cyclophosphamide (PT/Cy) strategies seem to improve survival by reducing GvHD incidence. Overall survival (OS) remains variable across groups. The first case presents rapid disease progression to pulmonary alveolar proteinosis (PAP) and leukemic transformation, further developing severe HSCT complications. The second case addresses novel *GATA2* mutation and raises concerns regarding alternative prophylactic and therapeutic strategies in transplant setting. **Conclusions:** Collaborative efforts aim to enhance understandings of *GATA2*-related myeloid malignancies and guide towards more effective management approaches.

## 1. Introduction

GATA binding protein 2 (*GATA2*) is one of the six GATA-family transcription factors, playing a key role in the development of the hematopoietic system. It is essential for hematopoietic stem cell survival and self-renewal, as well as for myeloid lineage differentiation and maintenance [1,2]. *GATA2* has been identified as a predisposition gene for familial myelodysplastic syndrome (MDS) with a high propensity to evolve into acute myeloid leukemia (AML). The phenotypic diversity resulting from the variable penetrance and expressivity of *GATA2* leads to clinical heterogeneity. This includes immunodeficiency linked to MonoMAC syndrome (monocytopenia, B/NK lymphopenia, and susceptibility to mycobacterial, fungal, or viral infections), lymphedema originally defined as Emberger syndrome, pulmonary alveolar proteinosis (PAP), and syndromic features such as sensorineural hearing loss or urogenital dysplasia [3,4]. Myeloid neoplasia occurs in approximately 75% of patients with *GATA2* mutations, primarily manifesting between the second and third decades of life, with minimal incidence in older individuals and secondary MDS [4,5].

This article highlights the complexities surrounding *GATA2*-related myeloid neoplasia in pediatric patients, addressing unresolved challenges in the current literature. To achieve this objective, we conducted a systematic review to evaluate the role of *GATA2* mutations in pediatric MDS/AML, focusing on disease characteristics, management strategies, and prognostic implications. The following section outlines the review methodology adhering to PRISMA (Preferred Reporting Items for Systematic Reviews and Meta-Analyses) guidelines. In the light of these findings, two challenging cases from the Pediatric Hematology and Oncology Department of Fundeni Clinical Institute, Bucharest, Romania, are further discussed.

## 2. Materials and Methods

### 2.1. Systematic Review

We conducted a systematic review following PRISMA guidelines [6] to evaluate the role of *GATA2* mutations in pediatric MDS/AML presented in open access articles published in international databases (PubMed, Scopus, Web of Science). The search strategy was developed using a combination of keywords related to “*GATA2* deficiency”, “*GATA2* mutation”, “pediatric *GATA2* related MDS/AML”, and “hematopoietic stem cell transplantation”.

Eligibility criteria included cohorts with over 10 patients, children and young adults (due to familial aggregation), diagnosed with *GATA2*-MDS/AML. Studies were required to address data on *GATA2* epidemiology, phenotype, disease management, and treatment. Only retrospective and prospective cohort studies, along with case–control studies published in English were considered.

**Figure 1:**
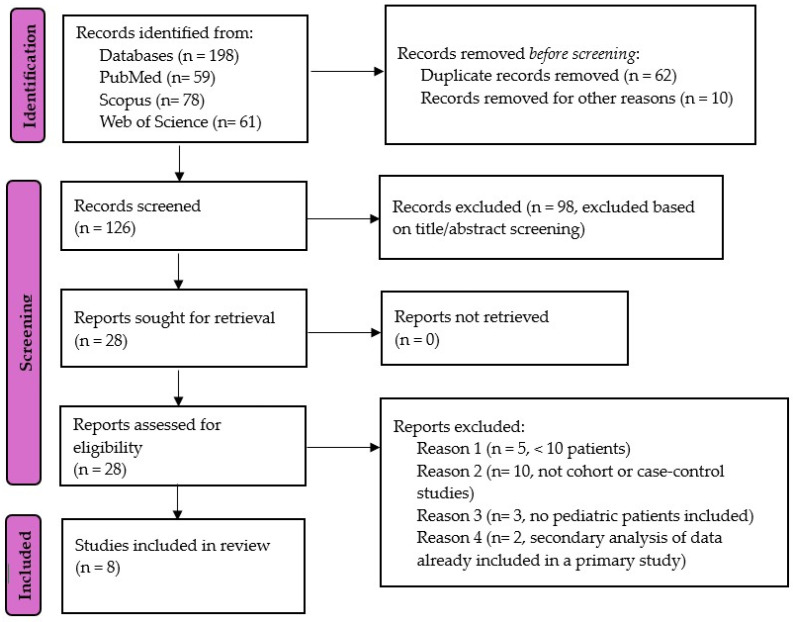
The PRISMA flow diagram.

Studies identified through literature searches were imported into EndNote 21.5, Clarivate Analytics, Philadelphia, PA, USA, where duplicates were removed. Screening titles and abstracts based on the established eligibility criteria were performed. Full-text articles were subsequently assessed and cross-checked for inclusion by two teams of reviewers. Studies which were not incorporated into the systematic review but provide relevant data are referenced for discussions. Following PRISMA guidelines, a systematic search identified 198 articles, of which 8 met the eligibility criteria, as shown in Figure 1.

We first collected information on authors, year of publication, and study design. Data extraction searched for details on disease characteristics and features, followed by outcomes (Table 1). Studies addressing hematopoietic stem cell transplantation (HSCT) management and complications, transplant-related toxicity (TRT) and mortality (TRM), as well as outcomes were synthetized separately (Table 2).

Risk of bias was assessed and cross-checked by two teams of reviewers, using the Newcastle–Ottawa Scale, with scores ≥8/9, demonstrating high validity. Critical analysis of study heterogeneity and inconsistencies was performed upon study design, age groups, key findings, follow-up time, and outcomes. Variability was addressed by highlighting the *GATA2*-MDS/AML subgroups, accounting for follow-up duration when analyzing outcomes, and mitigating reporting bias through separate data tables. The structured narrative approach facilitates a clear understanding of inter-study differences.

Despite efforts to ensure accuracy, the interpretations are subject to the limitations of the included studies. Readers should consider these limitations and refer to the original sources for further context.

Studies analyzed in Table 1 describe *GATA2* cohorts presenting with variable clinical and genetic characteristics. Median age at diagnosis ranges between 12.3 years [4] and 20 years [7], with a positive family history reported between 29% [4] and 67% of cases [5]. Disease subtypes commonly include monosomy 7 in 35% to 68% of cases [4,5], while trisomy 8 occurs at variable rates (9–24%). Recurrent infections are the most common clinical feature, with varying prevalence of immunodeficiencies, lymphedema, urogenital anomalies, and hearing loss. Prognosis worsens with age, as survival without HSCT declines from 89% at 20 years to 53% at 40 years [7]. While HSCT improves short-term survival (72–73% at 1 year), long-term outcomes decline to 54% at 4 years [7] and 62% at 5 years [5]. HSCT remains the primary treatment to prevent disease progression, emphasizing the need for continuous monitoring of infectious and hematologic complications.

Table 2 includes studies evaluating the main complications experienced by *GATA2* patients undergoing HSCT. Graft versus host disease (GvHD) incidence and severity are comparable between the *GATA2* cohort and control groups in one study [9]. The long follow-up of this study shows no differences in EFS and OS nearly 6 years after HSCT. Grade II-IV acute GvHD rates range from 34% [10] to 41.9% [12], while chronic GvHD varies from 9% [11] to 46% [8]. Post-transplant cyclophosphamide (PT/Cy) protocols significantly reduce GvHD incidence [8,11]. TRM remains variable (10–16.4%), often linked to GvHD and infections. OS differs across studies, with a 2-year OS of 86.4% [8] and 5-year OS ranging from 72.1% [12] to 93.3% [11], depending on donor type and conditioning regimen. Patients with *GATA2* mutations have higher risks of neurological, thrombotic, and infectious complications, with PT/Cy-based strategies showing promise in improving survival by reducing GvHD incidence.

Study inconsistencies arise from variability in design, cohorts, transplant protocols, and outcome reporting, hindering direct comparisons. While selected study cohorts vary in demographic composition, reflecting the diversity of *GATA2* disorders, we prioritized those focused on *GATA2*-MDS/AML. Some concentrate solely on pediatric patients [4,9,10], while others include both pediatric and adult populations [11,12]. HSCT studies vary in conditioning regimens (myeloablative vs. reduced intensity), GvHD prophylaxis (PT/Cy vs. anti-thymocyte globulin-based), and donor type (haploidentical vs. matched), influencing TRM, EFS, and OS. Disparities complicate cross-study comparisons, as higher-intensity regimens may elevate TRM independently of *GATA2* disease severity. Additionally, variations in outcome endpoints and follow-up duration introduce reporting bias, with shorter studies potentially overestimating TRM, while longer follow-ups may capture late relapses.

### 2.2. Case Presentations

We report two cases of children with *GATA2*-related MDS diagnosed in the Pediatric Hematology and Oncology Department of Fundeni Clinical Institute, Bucharest, Romania. Diagnosis and family germline testing was performed using standard protocols with support from the European Working Group on MDS in Childhood (EWOG-MDS), followed by monitoring, donor search, and treatment. The two case reports aim to emphasize the concerns *GATA2* MDS/AML determine in clinical practice. The first case underscores the aggressive progression from MDS to AML, highlighting the disease challenges and unique therapy-related complications that these patients experience. The second case addresses the prognosis and therapeutic complexities in *GATA2*, particularly when novel mutations with unknown functional consequences are identified. Despite the early HSCT, severe complications emerged, underscoring the need for better prophylactic and therapeutic strategies in *GATA2* patients. 

#### 2.2.1. Case 1: MDS-IB Associated with Germline *GATA2* Mutation and Monosomy 7, Developing Rapid Progression to MDS-Related Acute Myeloid Leukemia and Post-HSCT Complications

A 10-year-old girl presented with high fever, pneumonia with acute respiratory failure, and pancytopenia. Complete blood count (CBC) detected leukopenia (WBC 4.68 × 10^9^/L) with neutropenia (ANC 1.13 × 10^9^/L) and normal monocytes (AMC 0.57 × 10^9^/L), macrocytic anemia (Hb 6.6 g/dL, MCV 90 fL), thrombocytopenia (PLT 65 × 10^9^/L), and 10% blasts on peripheral blood smear. Complex hematological evaluation consisting of bone marrow aspirate (morphology, immunophenotyping, cytogenetics) and biopsy (see Table 3), confirmed MDS-IB with monosomy 7. Molecular analysis identified a pathogenic *GATA2* p.S201* mutation, predicted to truncate the *GATA2* protein at amino acid 201 of 480, upstream of the zinc finger domains, suggesting a loss-of-function effect. Secondary findings included mutations in *ASXL1*, *WT1*, and a second *GATA2* variant with an uncertain impact. Regarding additional testing, HbF analysis was not feasible due to red blood cell transfusion dependency. The immunological panel showed normal immunoglobulin levels and lymphocyte subset analysis identified reduced B- and NK-cells with a normal T-cell distribution.

The patient developed *Clostridium difficile* infection and severe respiratory infections overlapping pulmonary alveolar proteinosis (PAP), leading to mixed ventilatory disfunction chronic respiratory failure and oxygen dependency. Chest CT showed crazy paving, ground-glass opacities, and honeycomb-like changes. Hematological monitoring detected progression to MDS-related AML (MDR-AML) with 20% blasts in bone marrow with indication for intensive chemotherapy followed by HSCT. The treatment consisted of AML induction according to NOPHO-DBH-AML2012, with 0.04% MRD persistence, and low-dose cytarabine to maintain stable counts until HSCT.

The mother and brother tested negative for germline *GATA2* mutations. Family HLA typing did not show any compatible donor, and a search for an unrelated donor (UD) was initiated. Six months after diagnosis, the patient received HSCT from a fully matched 10/10 UD, CMV R+/D+, with a fresh graft of 5 × 10⁶ CD34+/kg peripheral blood stem cells (PBSC). Busulfan-melphalan conditioning (busulfan 3.2 mg/kg, no TDM for AUC available) was administered. GvHD prophylaxis included PT/Cy, tacrolimus, and MMF.

Post-HSCT early complications included grade II CRS, grade IV oral mucositis, and worsened pulmonary aspergillosis. Engraftment and 100% donor chimerism were achieved by day +20. The patient developed CMV disease, *Clostridium difficile* reactivation and steroid-refractory grade IV gut and skin GvHD despite intensive immunosuppressive therapy, including ruxolitinib, and extracorporeal photopheresis (ECP). Neurologic complications consisted of peripheral neuropathy, severe hyponatremia, and behavioral changes, specifically unexplained aggressiveness, voluntary dental extraction, and self-injury, which led to skin lesions (overlapping skin acute GvHD). Death occurred on day +46 post-HSCT.

#### 2.2.2. Case 2: Novel Germline *GATA2* Mutation Presenting as MDS-LB with Excessive Clinical Complications

A 9-year-old boy with a history of resolved asthma and no significant infections, was addressed for pancytopenia detected during routine blood tests. *CBC* detected severe leukopenia (WBC 1.38 × 10^9^/L) with neutropenia (ANC 0.47 × 10^9^/L) and low monocytes (AMC 0.08 × 10^9^/L), macrocytic anemia (Hb 9 g/dL, MCV 106 fL) and thrombocytopenia (PLT 44 × 10^9^/L). Complex hematological evaluation, consisting of bone marrow aspirate and biopsy, confirmed MDS-LB with no cytogenetic abnormalities (Table 3). A novel pathogenic *GATA2* p.Y377dup mutation was identified on molecular testing, causing a Tyrosine insertion at position 377. This mutation was not previously described in the literature but the bioinformatic analysis suggests this is a pathogenic mutation-MutPhred-Indel (score 0.86 and PPI hotspot (*p* = 0.04)) and mutation taster (prob: 0.999). This mutation is localized in the second zinc finger and is considered according to the ACMG criteria as a pathogenic variant (PM1, PM4, PM2). The germline nature of this mutation was further confirmed using hair follicle and Sanger sequencing. Pathogenicity of this variant is further confirmed by the clinical findings of this patient.

Secondary findings included a *MET* mutation and another *GATA2* (p.G346R) mutation, located between the two zinc fingers. While bioinformatic tools predict p.G346R as potentially damaging (FATHMM, PoliPhen2, SIFT, Mutation Taster), its impact remains uncertain due to the lack of biochemical characterization and literature reports. Additional testing showed increased 13.5% HbF, normal immunoglobulin levels, B and T lymphopenia with normal NK-cell subsets.

While allo-HSCT is the only curative treatment for *GATA2*-MDS patients, pre-transplant therapy is recommended during the search procedure, an epigenetic approach being a safe option, and azacytidine was initiated. BM surveillance identified an increase in blasts count (6%) and in the absence of a compatible donor, a haploidentical HSCT from his father (germline *GATA2*-negative) was recommended. Pre-transplant evaluation showed a normal pulmonary function test and 100% Lansky score. The patient received haploidentical transplantation from a 5/10 matched CMV D+/R+ donor, with 5 × 10^6^ CD_34_/kg PBSC fresh graft, after thiotepa-fludarabine-melphalan conditioning and GvHD prophylaxis with PT/Cy, tacrolimus, and MMF.

Early transplant complications involved grade III oral mucositis and *Clostridium Difficile* infection (CDI). The patient engrafted successfully, achieving 100% donor chimerism on day +21. The subsequent post-HSCT phase was complicated by recurrent CDI, regardless of various antibiotic regimens, including vancomycin, metronidazole, and fidaxomicin. He developed progressive grade IV acute gastrointestinal GvHD, corticosteroid-refractory, without response to ruxolitinib, ECP, and fecal transplantation. The patient developed severe malnutrition, peripheral polyneuropathy, and depression. His condition deteriorated due to sepsis, massive gastrointestinal bleeding, and secondary multiorgan failure, leading to death on day +90 post-transplantation.

## 3. Discussion

Germline *GATA2* mutations cause a phenotypically complex and variable spectrum of hematological manifestations, being the most common predisposing condition for childhood MDS [4,5]. Classically linked to monocytopenia, as seen in the second reported patient, *GATA2*-MDS can also present with monocytosis, especially in cases with monosomy 7 [4]. The first patient, however, exhibited a normal monocyte count, emphasizing the heterogonous nature of clinical presentation. This variability extends to immune disfunctions, as *GATA2*-MDS has been frequently associated with overlapping immunodeficiencies. B-cell loss is a key feature, often accompanied by other characteristics but less common manifestations such as NK lymphopenia [14]. Notably, the second patient exhibits a decreased T-cell population, presumably as part of a broader lymphopenia [15]. Investigating the genetic variants of *GATA2* and the mechanism behind T-cell dysregulation could provide insights into personalized treatments [16].

Given the potential impact on disease progression and treatment decision, timely *GATA2* assessment is critical for improving patient management and outcomes [7]. A 15-year EWOG study involving children with MDS showed that germline *GATA2* mutations account for approximately 15% of advanced and 7% of primary pediatric MDS. Of particular significance, these mutations are responsible for about two-thirds of adolescents with monosomy 7 and are absent in secondary MDS [4]. Although *GATA2* mutational status itself is not an independent prognostic factor, its impact increases when associated with advanced disease, cytogenetic abnormalities, or severe cytopenia [4,7,10].

Notably, neither of the two reported patients had prior clinical signs or family history of hematologic disease. This suggests that de novo mutations with prolonged latency may be involved [4,17]. Concerning mutation landscape, there are over 150 known *GATA2* mutations, which seem to have different functional implications [18]. Both Mir and Donadieu et al. reported that MDS/AML is highly linked to missense mutations, whereas deletions are associated with dysmorphic features and increased infection risks [5,19]. This complex mutational spectrum becomes even more challenging with novel mutations of unknown consequences. The p.Y377dup mutation described in the second case affected the zinc finger domain crucial for *GATA2* function, contributing to the hematological and immunological manifestations. The co-occurrence of an uncharacterized p.G346R variant complicates the genotype–phenotype correlation.

Secondary somatic mutations, such as *ASXL1* or RAS pathway mutations, indicate gene signatures of high-risk myeloid progression and poor prognosis [20,21,22,23]. The first case had an *ASXL1* N986S missense mutation that is not functionally characterized in the literature, prediction algorithms classifying it as likely benign [24]. However, the first patient experienced rapid disease progression. Throughout the literature, *ASXL1* is increasingly recognized as a significant “second hit” mutation in MDS, with nonsense and frameshift mutations occurring in 15–25% of cases being associated with poor prognosis [16,25]. A second finding identified a *WT1* R462Q mutation known to alter DNA binding properties [26]. Besides its roles in cell growth and differentiation, as both tumor suppressor and oncogene, *WT1* regulates dormancy of early progenitors and directs differentiation towards myeloid lineage [27]. Therefore, it is linked to poor prognosis in AML [28,29]. The second patient has a *MET* p.R988C mutation, with mixed evidence regarding its oncogenic potential, as functional studies are contradictory [30,31].

There are no standardized guidelines for managing *GATA2* mutations due to their high variability. HSCT in a timely manner is the only curative treatment that can reset hematopoiesis and reverse some *GATA2* effects [10]. Ideally, HSCT is to be performed during the hypocellular MDS stage, before progression to advanced MDS/AML [15,32]. Nevertheless, HSCT remains challenging by itself and especially in the presence of comorbidities such as invasive/invalidating infections or PAP [9].

When selecting a family donor, excluding germline *GATA2* is essential, as asymptomatic carriers are not rare [33]. Complete BM analysis is required for all potential family donors to rule out undiagnosed familial disease, even in the absence of known germline variants [34]. Once a suitable donor is identified, pre-HSCT management plays a crucial role in optimizing transplant outcomes [35]. The first patient received a single block of conventional chemotherapy to reduce blast counts, but subsequently experienced exacerbated toxic pulmonary complications and oxygen dependency.

*GATA2* deficiency resembles other inherited bone marrow failure syndromes in terms of high rates of transplant-related toxicity/mortality (TRT/TRM) after myeloablative regimens, due to cellular defects. This has not yet been systematically demonstrated, although some experts show TRM is independent of underlying *GATA2* deficiency [7,36]. MDS subtype is considered a more relevant prognostic factor, with relapse rates between 54% and 66% and higher in monosomy 7 [4,13]. While TRM and overall survival (OS) remain comparable to control groups, event-free survival (EFS) is reduced due to higher TRT [8,37]. Therefore, less toxic conditioning strategies are recommended for lower-risk patients, busulfan-cyclophosphamide-melphalan regimen being reserved only for high-risk patients [38,39].

Studies indicate that acute and chronic GvHD rates are similar between *GATA2* and control groups. However, PT/Cy significantly reduces severe GvHD without increasing relapse in myeloid diseases [10,11,40]. Given poor performance status, severe lung involvement, and immunodeficiency, the first patient received busulfan-melphalan with PT/Cy. ATG was omitted to minimize infection risk and viral reactivation. Although not a standard approach, this strategy covers the need for a strong, yet less toxic, myeloablative effect, while conferring GvHD control.

Despite PT/Cy prophylaxis, both patients experienced severe gastrointestinal GvHD, likely exacerbated by *Clostridium Difficile* infections (CDI), a known contributor to gut GvHD severity [41]. Their bidirectional interaction complicates management, increases TRM, and reduces OS [42].

Further HSCT-related complications reflect the broader spectrum of multisystemic non-hematologic complications seen in *GATA2* patients. Its role in specific tissues and cells is intensely studied. *GATA2* mutations disrupt endothelial-vascular integrity and cell adhesion, leading to coagulopathy and thrombotic events [13]. *GATA2* is also expressed in neural tissues, with studies reporting unique complications like peripheral polyneuropathy, noninfectious encephalopathy or unexplained behavioral changes, mainly as neurotoxicity after HSCT [4,7,37,43]. These neuropsychiatric manifestations, alongside the psychological burden of HSCT, align with our findings in the first reported patient, who exhibited unexplained aggressiveness, self-injury, and voluntary dental extraction. Such complications further underscore the need to investigate the role of *GATA2* in neural function and its potential contribution to neurobehavioral dysregulation post-HSCT.

PAP results from dysregulation of alveolar macrophage phagocytosis and inadequate clearance of surfactant proteins [7,44]. Patients have ventilatory defects (obstruction, restriction or mixed pattern) and structural anomalies on chest CT (reticular opacities, nodules, ground glass opacities, crazy paving para-septal emphysema, and subpleural blebbing) [15,25]. These complications often worsen after transplantation due to conditioning regimens and the specific proinflammatory status.

New strategies appear to be promising for managing advanced disease. Disruption of epigenetic regulation is a hallmark of MDS/AML. EZH2 expression and high trimethylation of histone H3 as an epigenetic regulator advocate for the use of azacitidine in these settings [45]. Concurrently, some groups studied BCL2 expression in *GATA2*-MDS patients and there is a clear link between apoptosis deregulation and disease progression that supports the use of venetoclax [46]. Waclawiczek et al. proposed the Mediators of Apoptosis Combinatorial (MAC) score, which incorporates the expression of the BCL2 family (BCL2, MCL1, and BCL-XL). An elevated MAC score provides strong rationale to use combined therapy azacitidine/venetoclax [33]. In addition, preemptive azacitidine and donor lymphocyte infusions after HSCT may help combat high relapse rates [8].

Despite variability in cohort composition, transplant protocols, and outcome reporting, our systematic review highlights the heterogeneous clinical course of *GATA2*-MDS/AML and reinforces HSCT as the cornerstone of treatment. The clinical complexity of these cases stems from the dual nature of the disease, a genetic syndrome and subsequent somatic mutations leading to MDS/AML. HSCT is effective for both specific features of genetic syndrome and for MDS/AML, but optimal timing is crucial, as early transplantation may improve survival and reduce MDS/AML risk. Given its invasiveness and high risk, HSCT should be cautiously considered for *GATA2* syndrome without hematologic malignancy, particularly when the clinical significance of mutations remains uncertain. Considering the rarity of these conditions, the two clinical cases promote international collaboration as imperative to advance disease recognition, facilitate early diagnosis, and establish standardized management frameworks to optimize long-term outcomes.

## 4. Conclusions

Identification of *GATA2* myeloid neoplasms requires a specific approach for management and therapeutic strategies. However, this condition is not yet included in any risk-scoring system. Joint efforts to systematize information aim to correlate real-world experience with all disease characteristics, therapeutic opportunities, potential adverse effects, and survival. Establishing future directions for potential targeted therapies are much awaited.

## Figures and Tables

**Table 1:** Characteristics and results of *GATA2* mutated (*GATA2*^mut^) MDS/AML studies.

First Author	Year	No. of Patients	Median Age	Family History	MDS Subtype	Karyotype *GATA2* Cohort	Other Features	Conclusions for *GATA2*^mut^ Group
**MA.** **Spinner [7]**	2014	Total, N = 57 ***GATA2*** **deficiency**	20 yo	30%	**MDS, N = 42** **AML, N = 7** CMML, N = 4	✓ 24% trisomy 8 ✓ 16% monosomy 7 ✓ 52% normal	✓ 82% major infections ✓ 86% B lymphocytopenia ✓ 82% NK lymphocytopenia ✓ 78% monocytopenia ✓ 53% NTM and warts ✓ 18% PAP ✓ 11% lymphedema ✓ 25% thrombotic events ✓ 33% hearing loss ✓ 14% hypothyroidism	✓ Symptom-free: 50% by age of 20, 25% by age of 30, 16% by age of 40 ✓ Survival time after onset: 91% by 5 years after onset, 84% by 10 years, and 67% by 20 years ✓ Survival without HSCT: 89% by age of 20, 76% by age of 30, and 53% by age of 40 ✓ HSCT (21 pts): 72% 1-year OS, 65% 2-years OS, 54% 4-years OS
**MW. Wlodarski [4]**	2016	Total, N = 508 Primary MDS, N = 426 Secondary MDS, N =82	12.3 yo	29%	RCC, N = 341 RAEB or RAEB-t, N = 85 ***GATA2*-MDS, N = 56**	✓ 68% monosomy 7 ✓ 7% del 7q ✓ 9% trisomy 8 ✓ 16% normal	✓ 51% infections ✓ 23% lymphedema ✓ 39% immunodeficiencies ✓ 19% behavioral changes ✓ 12% urogenital anomalies ✓ 9% deafness	✓ *GATA2*^mut^ OS inferior to *GATA2*^WT^ (73% vs. 84%, *p* < 0.05) ✓ HSCT (34 pts): 5-year OS (66% vs. 69%) and EFS (60% for both) between *GATA2*^mut^ vs. *GATA2*^WT^
**J. Donadieu [5]**	2018	Total, N = 79 **Germline *GATA2*^mut^** **patients**	18.6 yo	67%	**MDS, N = 55** **AML, N = 15** ALL, N =1 AA, N = 2 JMML, N = 1	✓ 35% complete or partial loss of chr 7 ✓ 18% trisomy 8 ✓ 12% other ✓ 35% normal	✓ 56% recurrent infections (viral, fungal, mycobacteria) ✓ 40% warts ✓ 15% lymphedema ✓ 3.8% PAP ✓ 56% bacterial infection ✓ 1.3% deafness ✓ 11% autoimmune features ✓ 5% urogenital anomalies	✓ Symptom-free: 38% at the age of 20, 8% at the age of 40 ✓ MDS/AL: 6% at the age of 10, 39% at the age of 20, 81% at the age of 40 ✓ Mortality: 6% at the age of 20, 42% at the age of 40, 69% at the age of 60 ✓ HSCT (28 pts): 73% 1-year OS, 62% 5-year OS

*yo = year-old, CMML = chronic myelomonocytic leukemia, NTM = non-tuberculous mycobacteria, PAP = pulmonary alveolar proteinosis, OS = overall survival, RCC = refractory cytopenia in childhood, RAEB = refractory anemia with excess blasts, RAEBt = refractory anemia with excess blasts in transformation, EFS = event-free survival, GATA2*^mut^ *= GATA2 mutated, GATA2*^WT^ *= GATA2 wild-type, ALL = acute lymphoblastic leukemia, AA = aplastic anemia, JMML = juvenile myelomonocytic leukemia, chr = chromosome, AL = acute leukemia, pts= patients.*

**Table 2:** Characteristics of the studies assessing HSCT in *GATA2* mutated (*GATA2*^mut^) cohorts including MDS/AML.

First Author	Year	No. of Patients	Median Age	GvHD	TRT	TRM	EFS/DFS/RFS	OS
**M.** **Parta** **[8]**	2018	Total, N = 22 ***GATA2*^mut^ MDS, N = 20 **	26 yo	✓ grade III-IV aGVHD: 26%MRD/URD 0% HRD ✓ cGvHD: 46%MRD/URD 28% HRD	✓ Three deaths in the URD group, none occurring in the first 100 days, indicating low regimen-related mortality	✓ 2 pts (9%) died in the URD group: 1 pt (GvHD) 1 pt (sepsis)	✓ 2-year DFS: 86% (according to type of donor)	✓ 2-year survival of the entire group: 86.4%
**I.** **Hofmann [9]**	2020	***GATA2* BMF/MDS/AL, N = 15** Cohort A (BMF/MDS), N = 25 Cohort B (AML/ALL), N = 40	15.7 yo	✓ Rates of acute and chronic GvHD were similar across groups	✓ 40% neurological toxicities ✓ 53% pre/post-HSCT thrombotic events ✓ higher rate of infectious or immunological complications	✓ No difference in TRM, infections, or GvHD among groups	✓ Lower EFS (7% ± 6%) compared to control A (28% ± 10%, *p* = 0.003) control B (33% ± 8%, *p* = 0.001)	✓ 5-year OS: 65% (similar across groups)
**R.** **Bortnick [10]**	2021	**Total, N = 65** ***GATA2*^mut^ MDS/AML**	12.8 yo	✓ aGvHD at 100 days: grade II–IV: 34% grade III–IV: 12% ✓ 24% developed cGvHD	✓ 20% pulmonary and liver toxicity ✓ 4.6% VOD, microangiopathy, GI complications ✓ 9.2% renal complications ✓ 6.1% neurological toxicities ✓ 3% cardiac complications ✓ 1.5% AIHA, pancreatitis	✓ Nine pts (13.8%) died of transplant-related causes	✓ DFS: 78% in *GATA2^wt^* 70% in *GATA2*^mut^	✓ 5-year OS: 82% in *GATA2^wt^* 75% in *GATA2*^mut^
**DX.** **Nichols-** **Vinueza [11]**	2022	**Total cohort *GATA2*^mut^, N = 59**	-	---	✓ Lower Bu, targeted AUC	✓ Six pts (10%) died	-	-
		MRD/URD Tacro/MTX, N = 19 MDS, N = 14	28 yo	✓ grade III-IV aGvHD: 32% ✓ cGvHD: 42%	✓ Cumulative Bu 72.6 mg*h/L	✓ 2 pts (GvHD) ✓ 1 pt (sepsis)	✓ 4-year EFS: 78.9%	✓ 4-year OS: 78.9%
		MRD/URD PT/Cy, N = 23 MDS, N = 14	29 yo	✓ Grade III-IV aGvHD: 0% ✓ cGvHD: 9%	✓ Cumulative Bu 63.1 mg*h/L	✓ 1 pt (graft failure) ✓ 1 pt (sepsis)	✓ 4-year EFS: 78.3%	✓ 4-year OS: 82.2%
		HRD PT/Cy, N = 17 MDS, N = 13	28 yo	✓ Grade III-IV aGvHD: 6% ✓ cGvHD: 24%	✓ HRD without cytogenetic anomalies: Cumulative Bu 33.3 mg*h/L ✓ HRD with cytogenetic anomalies: Cumulative Bu 49.2 mg*h/L	✓ 1 pt (immune reconstitution syndrome in 2nd HSCT)	✓ 4-year EFS: 88.2%	✓ 4-year OS: 93.3%
**FS.** **Fontbrune [12]**	2024	**Total *GATA2*^mut^ cohort, N = 67** MDS-LB, N = 44 MDS-IB, N = 10 AML, N = 13	17.4 yo (onset) 20.6 yo (HSCT)	✓ aGvHD at days 100: grade II–IV: 41.9% grade III–IV: 12.9% ✓ cGvHD: 1-year: 34.9% (severe 18.9%) 2-years: 41.6% (severe 23.3%)	✓ In patients transplanted with BM (N = 28, 42% of cohort), there was no toxicity-related death	✓ 11 pts (16.4%) died: 6 pts (aGvHD) 3 pts (infections) 1 pt (graft failure) 1 pt (cGvHD)	✓ 1-year RFS: 83.4% ✓ 2-year RSF: 76.8% ✓ 5-year RFS: 72.6%	✓ Total cohort 1-year OS: 83.4% 2-year OS: 76.8% 5-year OS: 72.1% ✓ MRD+MUD: 5-year OS: 89.6%

*BMF = bone marrow failure, AL = acute leukemia, TRT = transplant related toxicity, TRM = transplant related mortality, EFS = event-free survival, DFS = disease-free-survival, RFS = relapse-free survival, OS = overall survival, GATA2^wt^ = GATA2 wild-type, aGvHD = acute graft-versus-host-disease, cGvHD = chronic graft-versus-host-disease, VOD = veno-oclusive disease, AIHA = autoimmune hemolytic anemia, MRD = matched related donor, URD/MUD= matched unrelated donor, HRD = haploidentical donor, MDS-IB = MDS with increased blasts, MDS-LB = MDS with low blasts, Bu = busulfan, PT/Cy = post-transplant cyclophosphamide, BM = bone marrow, pt/pts = patient/s.*

**Table 3:** Case reports of two patients with *GATA2*^mut^ MDS-IB/LB.

	Case 1 (MDS-IB), Female					Case 2 (MDS-LB), Male				
**Morphological** **findings** **(including EWOG)**	**Dysplasia**		**Peripheral Blood**		**Bone Marrow**	**Dysplasia**		**Peripheral Blood**		**Bone Marrow**
	**Megakaryopoiesis/Platelets**		✓ giant platelets		-	**Megakaryopoiesis/ ** **Platelets**		✓ giant platelets		✓ micromegakaryocytes ✓ round separated nuclei ✓ binucleated nuclei ✓ severe dysplasia
	**Erythropoiesis/Erythrocytes**		✓ anisopoikilocytosis ✓ tear drop cells ✓ elliptocytes ✓ rare fragmentocytes		✓ hypoplasia	**Erythropoiesis/ ** **Erythrocytes**		✓ anisopoikilocytos ✓ elliptocytes ✓ ovalocytes ✓ microcytes		✓ double nuclei ✓ multinuclearity ✓ macrocytosis ✓ megaloblastic mat. ✓ cytoplasmatic bridging ✓ severe dysplasia
	**Myelopoiesis/** **Neutrophils**		✓ pseudo-Pelger cells ✓ vacuoles ✓ left shift		✓ vacuoles ✓ pseudo-Pelger-cells ✓ agranular cells ✓ left shift ✓ moderate dysplasia	**Myelopoiesis/ ** **Neutrophils**		-		✓ hypoplasia ✓ left shift ✓ pseudo-Pelger-cells
**BM blasts**	14% (flow-cytometry: CD45+ weak, CD34+, CD117+, HLADR+, CD38+, CD13+, CD33+, CD15−/+w, icMPO−/+w, CD7+					2% (flow-cytometry: CD34-, CD117++, HLADR++)				
**Karyotype/FISH**	46 XX/Monosomy 7 (first evaluation 46%, reevaluation 70%)					46 XY/No monosomy 7 or trisomy 8				
**Bone marrow ** **biopsy**	Erythroid and megakaryocytic dysplasia, 14–15% CD34+, 6–7% CD117+ Fibrosis					Cellularity ~80%, erythroid dysplasia with megaloblastic changes, megakaryocytic dysplasia, 3–4% CD34+				
**TruSight ** **Oncology 500 Panel**	**Gene**	**Depth**	**VAF**	**cDNA mutation**	**Protein mutation**	**Gene**	**Depth**	**VAF**	**cDNA mutation**	**Protein mutation**
	***GATA2*** *	3150	45%	c.599dupG	p.S201 *	***GATA2*** *	544	47.1%	c. 1129_1131dupTA C	p.Y377dup
	ASXL1 **	3121	50.9%	c.2957A>G	p.N986S	GATA2 **	824	7%	c.1036G>C	p.G346R
	WT1 **	1670	4.7%	c.1385G>A	p.R462Q	MET **	1892	45%	c.2962C>T	p.R988C
	GATA2 **	2171	12.7%	c.1349_1368del20	p.G450Dfs					
**EWOG-MDS**	*GATA2* exon 3 c.599dupG; [p.S201Ter], VAF 46%					*GATA2* exon 5 c.1129_1131dupTAC; [p.Y377dup], VAF 40%				
	Germline confirmation (hair follicles)					Germline confirmation (hair follicles)				
	Negative family screening					Negative family screening				
The variant classification system: * Tier I, strong clinical significance (level A and B evidence); ** Tier II, potential clinical significance (level C and D evidence) [13]										
